# P54 - Sex differences in the relation between BMI changes and the prevalence and severity of wheezing and asthma in the first year of life

**DOI:** 10.1186/2045-7022-4-S1-P109

**Published:** 2014-02-28

**Authors:** Gustavo Wandalsen, Leila Borges, Nathalia Barroso, Fabíola Suano, Javier Mallol, Dirceu Sole

**Affiliations:** 1 Federal University of Sao Paulo, Sao Paulo, Brazil; 2 USACH, Santiago, Chile

## Background

Rapid weight gain has been recently associated with asthma at school age, but its influence in respiratory symptoms during infancy is still unknown.

## Objective

To evaluate associations between changes in body mass index (BMI) with the prevalence and severity of wheezing and asthma in the first year of life.

## Methods

Answers to the International Study of Wheezing in Infants (EISL) questionnaire from 6541 parents living in six different cities of Brazil were analyzed. Data from reported weight and height at birth and at one year were used to calculate BMI (z scores). Rapid BMIz gain was defined by the difference superior to +1.0 and excessive by the difference superior to +2.0.

## Results

Rapid BMIz gain was found in 45.8% infants and excessive BMI gain in 24.4%. Boys showed a significantly higher BMIz gain than girls. Girls with rapid BMIz gain showed a significantly higher prevalence of hospitalization for wheezing (8.8% vs 6.4%; aOR: 1.4, 95%CI: 1.1 to 1.8), severe wheezing (18.1% vs 15.0%; aOR: 1.3, 95%CI: 1.0 to 1.5) and medical diagnosis of asthma (7.5% vs 5.7%; aOR: 1.3, 95%CI: 1.0 to 1.7). Girls with excessive BMIz gain also had a significantly higher prevalence of hospitalization for wheezing (9.8% vs 6.7%; aOR: 1.5, 95%CI: 1.1 to 2.0) and severe wheezing (18.9% vs 15.5%; aOR: 1.3, 95%CI: 1.0 to 1.6). No significant association was found among boys. Breastfeeding was significantly less frequent among infants with rapid and excessive BMIz gain.

## Conclusions

The majority of the evaluated infants showed BMIz gain above expected in the first year of life. Although more commonly found in boys, rapid and excessive BMIz gain in the first year of life were significantly associated with more severe patterns of wheezing in infancy only among girls.

